# Reconstruction of oral mucosal defects using the nasolabial flap: clinical experience with 22 patients

**DOI:** 10.1186/1758-3284-3-28

**Published:** 2011-05-23

**Authors:** Andre M Eckardt, Horst Kokemüller, Frank Tavassol, Nils-Claudius Gellrich

**Affiliations:** 1 Department of Cranio-Maxillofacial Surgery, Hannover Medical School, Carl-Neuberg-Strasse 1, 30625 Hannover, Germany

**Keywords:** nasolabial flap, intraoral reconstruction, local flap

## Abstract

**Background:**

Various surgical options are available for reconstruction of intraoral soft tissue defects. For smaller defects of the oral mucosa in different anatomic locations of the oral cavity the nasolabial flap is a very useful and simple alternative to other pedicled flaps and free flaps.

**Methods:**

The results of reconstruction of oral mucosal defects or facial skin defects using 29 nasolabial flaps in 22 patients were reviewed retrospectively.

**Results:**

The patient group consisted of 16 patients (70%) with squamous cell carcinoma of the oral cavity, 2 patients (10%) with cystic lesions of the maxilla, 3 patients (15%) with osteonecrosis of the jaw, and 1 patient with an oral metastasis of a lung carcinoma. Healing was uneventful in 93%, partial or complete flap loss was observed in 7%.

**Conclusions:**

The nasolabial flap is a valuable alternative for reconstruction of smaller defects of the oral cavity in particular in older and medically compromised patients.

## Introduction

Mucosal defects in the oral cavity as a result of tumor resection, acute or chronic infections may leave the patient with a significant functional and esthetic defect. As far as tumor resection is concerned the likelihood of postoperative functional impairment is clearly related to the anatomic site of the tumor as well as size of the tumor. T1 tumor lesions usually do not cause a problem and primary closure of the mucosal defect is the treatment of choice in most cases. However, defects from resection of T2 tumors are often too large for local closure and require distant or local flaps. A variety of regional cutaneous and myocutaneous flaps from various donor sites are available and of course in complex defects microvascular free tissue transfer from distant sites is a well accepted surgical option [1,2]. Such surgical procedures however may not be indicated in medically compromised patients [2].

The nasolabial flap is an arterialized local flap in the head and neck region with an axial blood supply provided either by the facial artery (inferiorly based) or by the superficial temporal artery through its transverse facial branch and the infraorbital artery (superiorly based) [3]. It is a reliable, versatile, and easy to raise flap for a variety of small to intermediate defects in the orofacial region. The first nasolabial flap for intraoral reconstruction was reported at the end of the nineteenth century [4,5]. Superiorly based nasolabial flaps can be used for reconstruction of nasal defects, lower eyelid, and the cheek, whereas the inferiorly based flaps are considered useful in reconstruction of defects of the lip, oral commissure, and the anterior oral cavity [6].

This paper presents our clinical experience with nasolabial flaps for reconstruction of mucosal defects due to ablative tumor surgery or mucosal breakdown in acute or chronic infections.

## Patients and methods

From 2004 to 2010, a group of 22 patients underwent reconstruction of intraoral mucosal defects using a nasolabial flap. A retrospective data analysis was performed using data from patient medical records, including data on underlying pathology, defect size and location, surgical technique of flap harvesting.

## Results

Relevant demographic patient data are listed in Table 1. There were 15 male and 7 female patients, with a mean age of 67 years (range 49-85 years). In 16 patients (73%) the underlying pathology was squamous cell carcinoma, in 3 patients (14%) the nasolabial flap was used to cover mucosal defects of the lower alveolus after resection of osteoradionecrosis (1 patient), chronic osteomyelitis (1 patient), or the upper alveolus after resection of bisphosphonate-related osteonecrosis (1 patient). Two patients (9%) had extensive cystic lesions of the maxilla and flaps covered mucosal defects of the upper alveolus, and one patients was diagnosed with an intraoral metastasis of a lung carcinoma. In 7 of 16 tumor patients (43%) the floor of mouth was involved, whereas the lower or upper alveolus was involved in the remaining 9 patients (56%). Patients diagnosed with oral squamous cell carcinoma (n = 16) underwent transoral tumor resection, supraomohyoidal neck dissection and simultaneous reconstruction with a nasolabial flap.

**Table 1:** Characteristics of patients with intraoral nasolabial flap reconstruction (n = 22)

	N	%
Gender		
Male	15	68
Female	7	32

Age, years		
Mean	67,7	
Range	49-85	

Disease		
Squamous cell carcinoma	16	73
Osteonecrosis of bone	3	14
Extensive maxillary cyst	2	9
Metastasis of lung carcinoma	1	4

Stage of tumor (n = 16)		
pT1pN0	6	37,5
pT2pN0	4	25
pT2pN1	2	12,5
pT2pN2a	1	6,25
pT4pN1	3	18,75

Donor site		
Left	10	45
Right	5	23
Bilateral	7	32

Defect location		
Lower alveolus	8	36
Upper alveolus	7	32
Floor of mouth	7	32

Flap orientation		
Inferiorly based	19	10
Superiorly based	66	34

Nasolabial flaps were raised unilaterally in 15 patients, bilaterally in 7 patients, comprising a total of 29 flaps. All flaps were performed as a single-stage procedure with 66% inferiorly-based flaps. The orientation of the pedicle is usually determined by the location of the defect and the requirements of rotation and advancement of the tissues to the defect. An inferiorly based flap is outlined in the cheek with the tip of the flap situated caudally to the medial canthus depending on the required length of the flap. The flap base is situated little below or just above the angle of the mouth. This design allows a flap length of 5-7 cm. With a width of the flap of up to 3 cm, the donor site can be closed primarily without tension. The flap is dissected in a supramuscular plane, keeping the base of the flap as thick as possible. Entrance to the oral cavity is achieved by dissecting a transbuccal tunnel situated just opposite to the oral defect. Care must be taken not to injure the parotid duct while dissecting the tunnel. Also sufficient width of the tunnel is necessary to avoid constriction of the pedicle. Those parts of the flap pedicle which are placed in the tunnel need careful de-epithelialization. Finally the skin island covering the intraoral defect is carefully sutured into its definitive position. In most cases neither pedicle transsection nor debulking of the intraoral skin island is required. Median operation time for the whole procedure was 170 minutes (range 70 - 530 minutes). Uneventful healing was observed in 93% of the flaps (n = 27), partial or complete flap loss was observed in 7% (n = 2). In only 1 out of 29 flaps (3%) we observed problems with infection and inclusion cysts after de-epithelization of the pedicle. Follow-up ranged from 1 month to 5 years (mean 3.1 years).

### Case reports

#### Case 1

**Figure 1:**
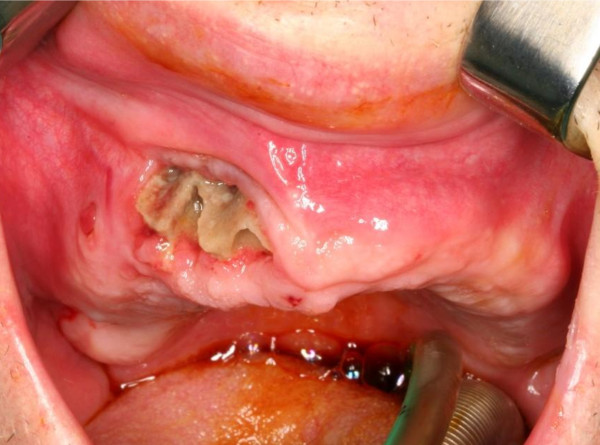
Intraoral aspect of a bisphosphonate-associated osteonecrosis of the maxilla (Stage 2) in a patient suffering from a metastasizing carcinoma of the prostate.

**Figure 2:**
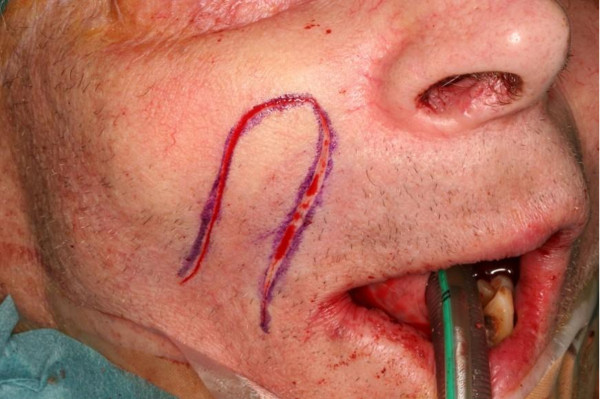
**Typical outline of an inferiorly based nasolabial flap for intraoral defect closure **. The flap is raised in a supramuscular plane of dissection, partially de-epithelialized in preparation for single-stage transfer.

**Figure 3:**
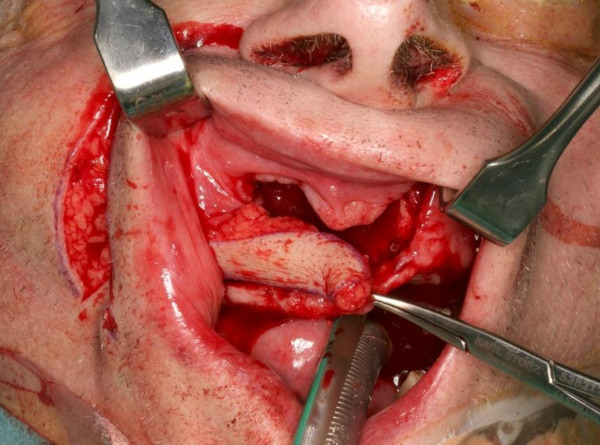
**The flap and the subcutaneous pedicle have been elevated and following dissection of a transbuccal tunnel the flap is ready for "pull-through" and inset into the anterior maxillary defect **.

**Figure 4:**
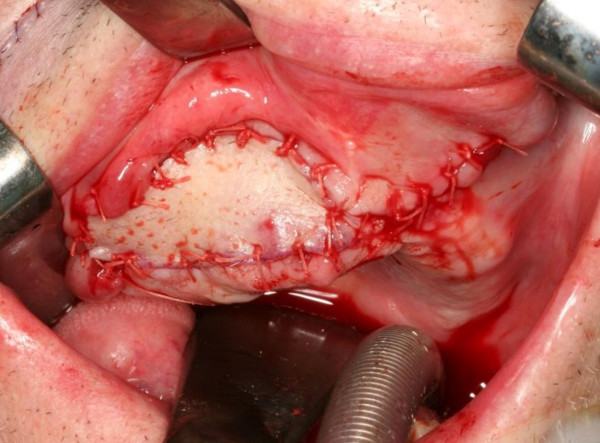
**Clinical appearance of nasolabial flap after inset to the anterior defect of the maxilla**. The flap margins are sutered to the mucosa in a single-stage procedure.

**Figure 5:**
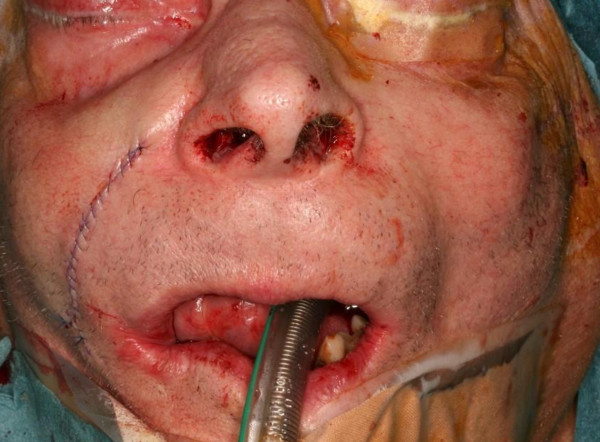
**Donor-site area in the nasolabial fold is closed in a tension-free manner**. No facial asymmetry is noticed. On a longterm perspective the nasolabial scar is minimally noticeable.

A 76-year old male patient suffered from a metastasizing carcinoma of the prostate. As part of his oncologic treatment he received i.v. zolendronic acid (Zometa^® ^) on a monthly basis for the last 8 months. Following dental extractions of his upper jaw he developed bisphosphonate-related osteonecrosis which didn't respond to conservative treatment. On intraoral examination a 2 × 1 cm area of exposed necrotic bone was visible in the right anterior maxilla (Figure 1). In view of impaired mucosal healing in patients receiving i.v. bisphosphonates it was decided to provide a well vascularized soft tissue closure with a local flap after partial resection of the anterior maxilla. Resection of necrotic bone and reconstruction of the mucosal defect with a nasolabial flap was performed under general anesthesia. An inferiorly based nasolabial flap was raised, a transbuccal tunnel was dissected and flap inset was achieved following partial de-epitheliazation (Figure 2, 3). The donor site was closed primarily and the intraoral skin island provided adequate coverage of the former necrotic bone area (Figure 4, 5). The patient showed up for monthly follow-up visits for another 13 months.

#### Case 2

**Figure 6:**
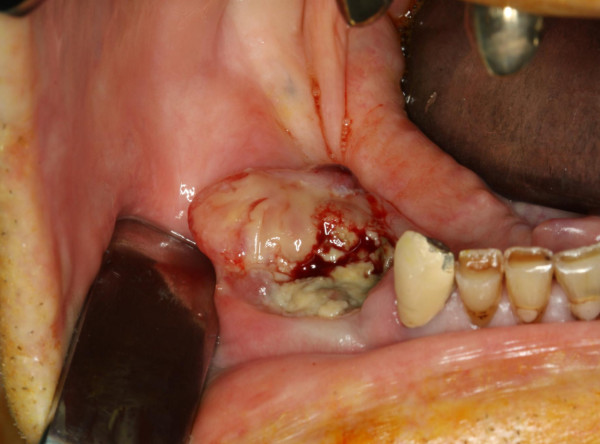
**Rapidly tumor mass of the lower alveolus histologically diagnosed as intraoral metastasis of a lung carcinoma**. Due to rapid tumor growth local resection with palliative intention was selected as treatment of first choice.

**Figure 7:**
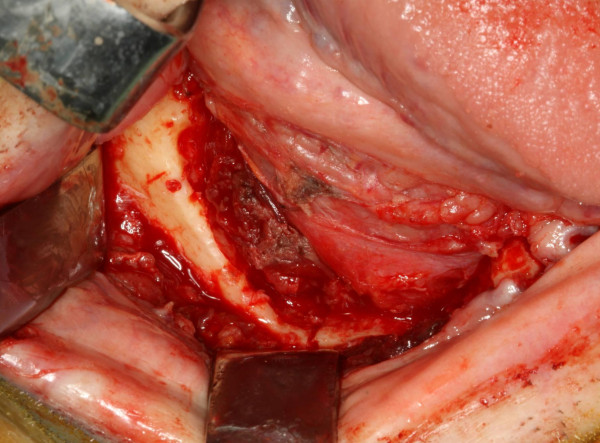
Intraoral defect of 3 × 3,5 cm following transoral mandibular resection of the metastasis.

**Figure 8:**
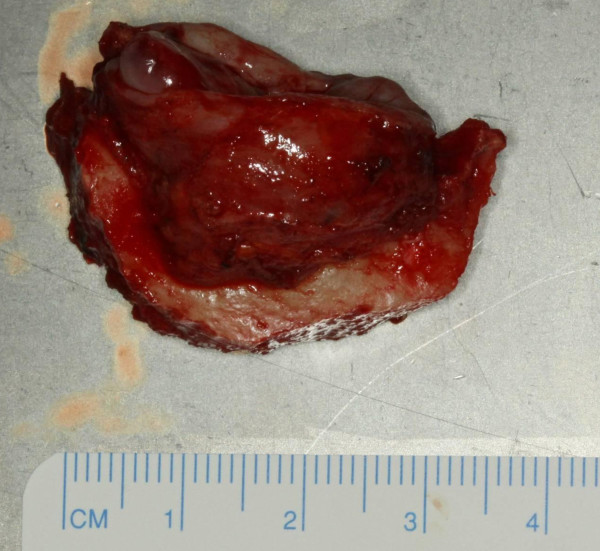
Figure 8 shows mandibular resection specimen. Frozen section of resections margins revealed no tumor infiltration.

**Figure 9:**
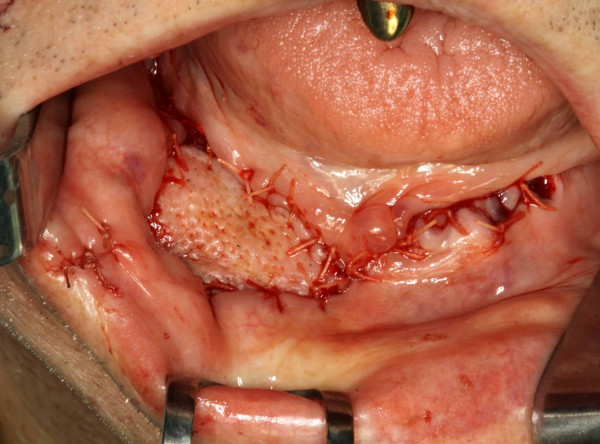
Intraoral view after defect closure with an inferiorly based nasolabial flap shows well perfused skin island.

A 70-year old male patient suffered from a metastasizing lung carcinoma. He was referred to our institution because of a rapidly growing mass of the lower right alveolus which was histologically diagnosed as intraoral metastasis of a lung carcinoma (Figure 6). Transoral resection of the metastasis with marginal mandibulectomy was performed under general anesthesia (Figure 7, 8). In the same procedure the defect was closed with an inferiorly based nasolabial flap (Figure 9). The patient continues to be followed-up by the oncologic service.

## Discussion

Reconstruction options for smaller defects of the oral cavity are ranging from primary closure, secondary healing from mucosalisation, or covering the defect site with split thickness skin grafts. Most of these techniques may result in speech and swallowing problems. Intraoral reconstruction with the nasolabial flap is a simple and fast procedure and minimizes the morbidity relating to speech and swallowing impairment to a great extent [3,7]. Adequate oral function and esthetic results following reconstruction of smaller defects of the anterior floor of mouth were confirmed by Hofstra et al (2004) [8]. In their series of 26 patients with intraoral reconstruction using a nasolabial flap, Maurer et al (2002) reported that 23 patients (88%) underwent successful prosthetic rehabilitation and they concluded that the nasolabial flap is a functional and esthetically satisfactory alternative compared to free tissue transfer [9]. However, the bulkiness of the inferiorly based nasolabial flap may be a disadvantage and may cause some difficulties in wearing dentures [7]. Although the nasolabial flap has been initially used for reconstruction for nasal and facial skin defects [4,5] there is nowadays well documented evidence that this flap can be ideally used for reconstruction of smaller defects in the oral cavity [10-22]. The reported defect size ranged from small defects (2-4 cm) to moderate defects (4-6 cm) [3,7,9]. Varghese et al. (2001) published the largest series of nasolabial flaps for intraoral reconstruction with 224 patients [3]. An inferiorly based nasolabial flap was used in 198 patients, whereas 24 patients were reconstructed using an superiorly based flap. The authors reported significantly more complications in post-irradiated cases than in primary cases (p = 0.03). In contrast, van Wijk et al (2000) did not find a correlation between flap survival and radiotherapy and relates this mainly to the excellent vascularity of the nasolabial flap [7]. The complication rate of nasolabial flaps in general is low. Varghese et al. (2001) reported of a flap loss rate of 5.5% (partial loss) and 6.3% (complete loss) respectively in their series of 238 patients [3]. Comparable results with a partial flap loss of 5% were reported by van Wijk et al. (2000) [7]. Maurer et al. (2002) had no flap loss in their series, but they reported on wound healing problems in 11% which healed under conservative treatment [9]. With an overall flap loss rate of 7% in our patient group, our results are in the range of other published flap loss rates. Even in cases who had undergone neck dissection with simultaneous dissection of a nasolabial flap on the same side, no adverse effects on blood supply of the flap were observed [7,22]. This in fact corresponds to the assumption that not only the facial artery supplies the flap, but probably a rich subdermal plexus [23]. Based on our own experience with 16 patients with oral squamous cell carcinoma, simultaneous supraomohyoidal neck dissection has no negative effects on nasolabial flap healing and survival.

The use of nasolabial flaps in patients with limited defects of the anterior floor of mouth after tumor resection showed adequate functional and esthetic results [8]. Intraoral reconstruction using nasolabial flaps is a simple and fast procedure and can be recommended in particular in patients with medical comorbidities who are not candidates for time-consuming operations including microsurgical reconstructions.

## Conclusions

Based on published reports as well as our own experience we came to the conclusion that the nasolabial flap has proved to be a useful and reliable alternative for smaller to medium size defects of the oral cavity in order to allow wound closure without tension and maintain oral function.

## Competing interests

The authors declare that they have no competing interests.

## Authors' contributions

AME made substantial contributions to conception and design of the manuscript as well as data acquisition.

HK, FT have been involved in drafting the manuscript.

NCG was involved in revising the manuscript. All authors read and approved the final manuscript.
